# Mitigation of radiation mediated testicular dysfunction by α-tocopheryl succinate: PPAR-γ related pathway

**DOI:** 10.1186/s12906-025-04950-7

**Published:** 2025-06-09

**Authors:** Heba A. Gheita, Ghada M. Shafey, Maha M. Aziz, Noha A. Fadel

**Affiliations:** Drug Radiation Research Department, National Centre for Radiation Research and Technology, Egyptian Atomic Energy Authority (EAEA), Cairo, Egypt

**Keywords:** Radiation, Testicular dysfunction, α-tocopheryl succinate, PPAR-γ

## Abstract

**Background:**

Radiation exposure of sensitive organs during radiotherapy merits extraordinary consideration, particularly when the concern is about fertility. Although alpha-tocopheryl (vitamin E) is a potent antioxidant, many studies have demonstrated the radioprotective impact of alpha-tocopheryl acetate ester, emphasizing its antioxidant and anti-apoptotic effects; fewer studies were conducted using the succinate ester without any declaration of its anti-inflammatory effect in the concerned pathology. Accordingly, the current study was conducted to evaluate the dual antioxidant and anti-inflammatory role of alpha-tocopheryl succinate (α-TCS) in reproductive toxicity induced by gamma-irradiation.

**Methods:**

Animals were subjected to 6 Gy of whole-body gamma radiation and received α-TCS (200 mg/kg, P.O.) pre- and post-radiation. After the termination of the experiment, serum testosterone was estimated, and the testis weight was recorded. Besides, the testicular content of oxidative balance markers [malondialdehyde (MDA), superoxide dismutase (SOD), catalase (CAT)] and inflammatory response markers [interleukin-1β (IL-1β), nuclear factor-kappa B (NF-κB) p65, peroxisome proliferator-activated receptor-γ (PPAR-γ)] were assessed.

**Results:**

Irradiated (IR) rats showed disturbances in the testicular function and abnormal incidental lesions, as demonstrated in the histopathological examinations. They exhibited marked alterations in the testicular oxidative balance, verified by the rise of lipid peroxidation end product (MDA) and depletion of antioxidant enzymes (CAT and SOD). Also, radiation exposure triggered an inflammatory response, which was evidenced by suppression of PPAR-γ and intensified expression of NF-κB p65 subunit, with subsequent elevation in IL-1β testicular content. Conversely, administering α-TCS to IR rats maintained the testicular architecture and ultrastructure while also preserving testicular function. Treatment with α-TCS restored the oxidative balance (MDA, SOD and CAT) and reduced testicular content of pro-inflammatory cytokine IL-1β via interference with the NF-κB p65/ PPAR-γ signaling pathway.

**Conclusions:**

The current study sheds light on the crucial radioprotective role of α-TCS as a PPAR-γ agonist in maintaining testicular function partially through suppressing NF-κB activation and its downstream pro-inflammatory mediators.

## Introduction

The correlation between radiation exposure and maintenance of normal testicular and reproductive function is one of the most challenging goals for a radiation oncologist, especially when treating a patient of a younger age. Irradiation of the testis can occur during therapeutic and diagnostic medical procedures with two possible clinical scenarios: direct testis irradiation or scattered irradiation [1]. Testis direct irradiation occurs in several cases, like bone marrow transplantation, which requires regimens that commonly involve whole body gamma-irradiation (WBI). Most patients receiving WBI developed gonadal failure. On the other side, testis may be exposed to scattered radiation (1.5–3 Gy) during the treatment of some types of tumors, which involve cancer of the prostate, rectum, bladder, or lymphoma [1].

The ability of radiation to harm gonadal tissue has been extensively verified in various experimental studies [2–4]. The deleterious effect of radiation on germ cells is clear even at very low dose rates, demonstrated by clusters of apoptotic gonocytes [4]. Exposure to high dose-rate radiation negatively influences cell proliferation, leading to death and depletion of spermatogonia cells, including their subsequent generations, thereby adversely impacting testis weight [5]. Morover, radiation has been shown to trigger apoptosis in the seminiferous tubules [6] and to induce degeneration of spermatogonia, spermatocytes, and spermatids in the seminiferous tubules, which in turn caused severe testicular atrophy [7].

Initially, the mechanistic technique of radiation-induced normal tissue injury involves the production of reactive oxygen species (ROS), which surpasses the antioxidant system's capability and causes oxidative damage to proteins, lipids, and DNA [8]. Besides oxidative stress, the early, sustained, and exaggerated inflammatory reactions that were triggered following irradiation played a crucial role in the exacerbation of the deleterious effects [9]. In this context, one of the initiating factors that are highly implicated in the radiation injury is down-regulation of peroxisome proliferator-activated receptor (PPARs). It is a member of the nuclear hormone receptor superfamily, which regulates the expression of numerous target genes and several metabolic processes [10, 11]. The major role of PPAR is to induce inactivation of nuclear factor-kappa B (NF-κB), a transcription factor which binds its target genes and induces transcription of a wide array of inflammatory cytokines such as tumor necrosis factor-alpha (TNF-α) and interleukin-1β (IL-1β). PPAR binds directly to NF-κβ and induces its inactivation via ubiquitination, aiming to reduce the pro-inflammatory response. Additionally, PPAR exerts an oblique indirect impact on NF-κB by stimulating the expression of antioxidant enzymes like catalase (CAT), superoxide dismutase (SOD), or heme oxygenase-1, leading to diminution of ROS, which are secondary transmitters in the inflammatory response [12]. Radiation exposure has been proven to reduce PPAR expression, which in turn activates NF-κB, leading to development and exacerbation of inflammation [7, 13]. Therefore, modulating the PPAR/NF-κB axis was considered a crucial therapeutic target for effective radioprotectors.

Alpha-tocopheryl (vitamin E) is the most lipid-soluble antioxidant present in all cell membranes [14]. A wide array of studies have shown the radioprotective potential of alpha-tocopheryl on the male reproductive system, focusing on its antioxidant and anti-apoptotic properties without exploring its possible anti-inflammatory impact in various pathways [15–18]. For instance, vitamin E has been shown to counteract the detrimental effects of oxidative stress on testicular function, as it improves the signs of semen quality (sperm count, motility, and normal morphology) and restores normal testosterone level in nicotine- [15] and deltamethrin- [16] treated rats. Also, it promotes spermatogenesis by regulating the expression of proteins associated with plasma membranes and protamine biosynthesis [17]. Moreover, vitamin E was capable of hindering the apoptotic pathway in cadmium-induced testicular damage [18] and improving morphometrical measurements of seminiferous tubules in sodium arsenite-treated rats [19]. Additionally, vitamin E has been shown to antagonize the reproductive endocrine toxicity and to alleviate the induced deteriorations in testicular structure [20]. Apart from the studies stated above, most studies have employed the alpha-tocopheryl conjugated with acetate ester form; only few studies were conducted using the succinate ester [21, 22] and have not disclosed its potential function in hindering the inflammatory cascade in the concerned pathology. Compared to alpha-tocopheryl acetate, alpha-tocopheryl succinate (α-TCS) showed patent antioxidant as well as anti-inflammatory activities [23, 24].

In light of all the aforementioned background, the current study was conducted with the hypothesis that α-TCS would mitigate the radiation-induced testicular injury. Thereafter, to gain further insight into the molecular mechanisms, we investigated α-TCS’s potential on testicular oxidative stress and inflammation with a particular focus on PPAR-γ/NF-κB related pathway.

## Materials and methods

### Experimental animals

Eight-week-old male mature Sprague–Dawley rats (150 − 200 g) were employed in this investigation. Animals were obtained from the National Centre for Radiation Research and Technology (NCRRT, Cairo, Egypt). Rats were kept in air-conditioned quarters at a temperature of 25 °C with 12-h cycles of darkness and light. Animals were acclimated for one week before doing any trials. Rats were fed standard diet pellets containing a minimum of 5% fibre, 20% protein, 3.5% fat, 6.5% ash, and a vitamin blend. They were given unlimited access to water. After decapitation, animal carcasses were disposed of by burial. All animal experiments were conducted in compliance with the ARRIVE guidelines and were carried out following the U.K. Animals (Scientific Procedures) Act, 1986 and associated guidelines, EU Directive 2010/63/EU for animal experiments. The in vivo study and all the methods were performed according to the guidelines set by the Research Ethics Committee of the National Centre for Radiation Research and Technology (REC-NCRRT), Cairo, Egypt (permit number: 1 A/22).

### Irradiation

Whole body gamma-irradiation was performed at a dose level of 6 Gy for the induction of testicular damage [25]. Briefly, a cesium (^137^Cs) source was used in the Gamma Cell-40 biological irradiator at the NCRRT in Cairo, Egypt, to expose subjects to gamma rays. The animals were kept in cages with good ventilation and placed in a chamber that was attached to the irradiation apparatus. The animals were placed in a field of approximately 25X25 cm^2^ with a distance of 70 cm from the source, and they were subjected to a single dosage of 6 Gy at a rate of 0.47 Gy/min.

### Alpha-tocopheryl succinate preparation and administration

α-TCS was obtained from Solgar, Inc. (Leonia, NJ, USA) and dissolved in olive oil (Manfredi Barbera and Figli SPA, Italy) [26]. α-TCS was administered orally to rats at a dose of 200 mg/kg. The dose of α-TCS was selected based on earlier studies that confirmed its efficacy in reproductive toxicity and testicular oxidative stress [27, 28]. Moreover, the treatment period was conducted in compliance with the irradiation regimen that had been used for the induction of testicular damage [25].

### Experimental design

Animals were allocated into four groups (*n* = 6) and treated as follows:

- Group I (Normal group): rats received distilled water and served as normal control.
- Group II (IR group): rats received distilled water, and they were exposed to WBI at a dosage level of 6 Gy.
- Group III (vehicle + IR): rats received olive oil (5 ml/kg, P.O.) once daily. Then, they were subjected to WBI at a dosage level of 6 Gy.
- Group IV (α-TCS + IR): rats received α-TCS (200 mg/kg, P.O.) dissolved in olive oil once daily. Then, they were exposed to WBI at a dosage level of 6 Gy.

**Fig. 1:**
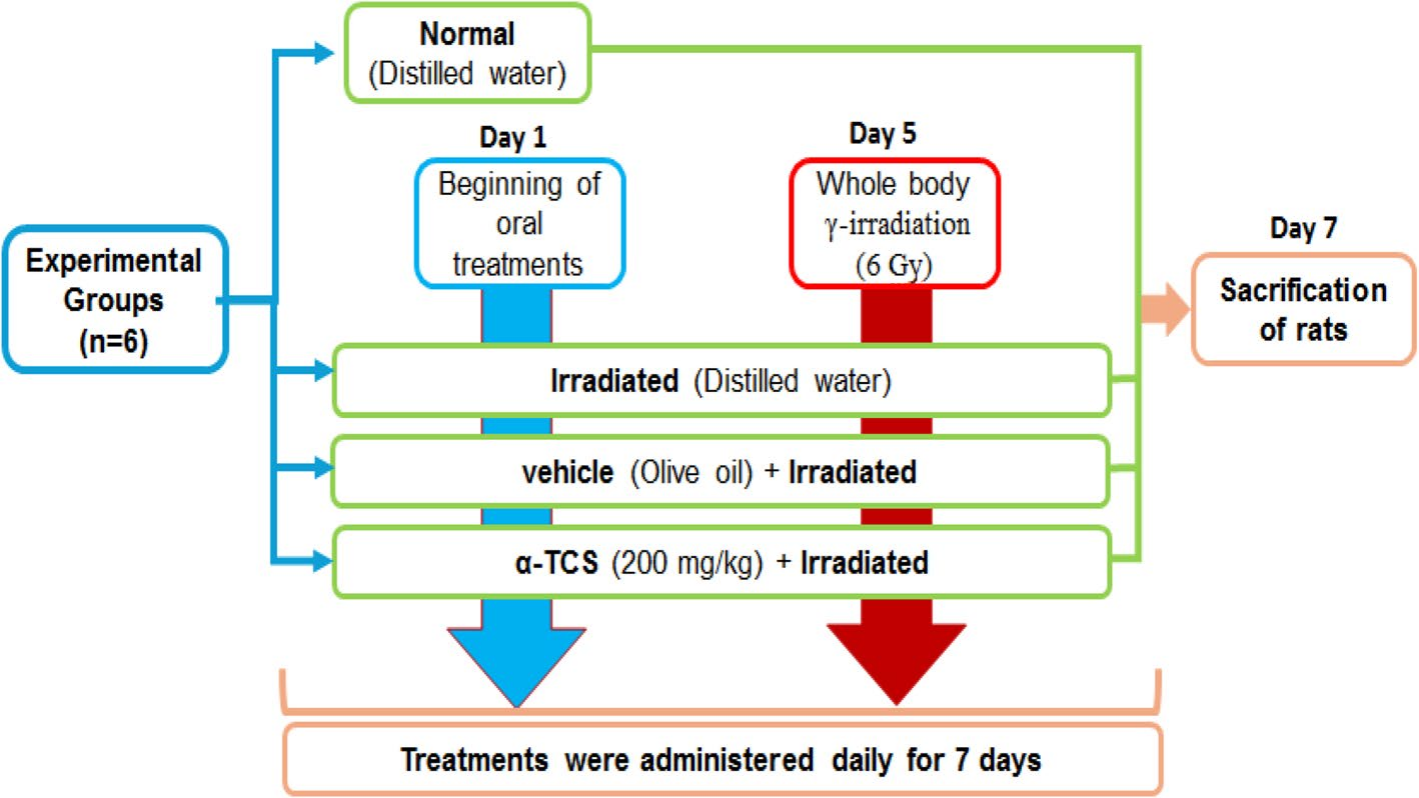
Workflow diagram of experimental design

The experiment was conducted on day 1 and lasted for 7 days. Treatments were administered once daily for seven days, and irradiation of rats was carried out on the fifth day [25]. On the seventh day after the last dosage by 6 h, all rats were anesthetized with urethane (1.2 g/kg, i.p) [29], and then sacrificed by cervical dislocation. A workflow diagram depicting the experimental design is presented in Fig. 1.

Blood samples were obtained, and serum was isolated by centrifugation at 3000 rpm for 15 min. Afterwards, testis tissues were dissected, washed with ice-cold saline, weighed, and then homogenized in ice-cold phosphate buffered saline using ultra-turrax tissue homogenizer to prepare tissue homogenate (20% weight/volume). The homogenized tissue was centrifuged for 15 min at 3000 rpm, and the supernatant was kept at −80 ◦C for further estimation of biochemical parameters. Additionally, more testicular tissues were fixed in formalin for histopathological and immuno-histochemical examinations.

### Estimation of serum testosterone and relative testis body weight ratio

Serum testosterone level was determined by using Enzyme-linked immunosorbent assay (ELISA) technique using rat-specific testosterone kit (Cat# E-EL-0477, Elabscience®, Texas, USA) according to the manufacturer’s instructions. Briefly, samples, standard, and blank were added to a 96-well plate pre-coated with antibody specific for rat testosterone. Following that, the biotinylated detection antibody specific for testosterone and Avidin-Horseradish Peroxidase (HRP) were added successively to each well, interspersed with a washing phase. After washing away any free components, the substrate solution was pipetted and the color developed proportionally to the amount of protein bound. The enzyme–substrate reaction is terminated by the addition of stop solution. The absorbance (OD value) was measured at 450 nm using a microplate reader, Dynatec MR5000 (Guernsey, Channel Islands, UK). Serial dilutions of known standard concentrations were used to create a standard curve, and the concentration was calculated accordingly. Results are expressed as (ng/ml), the intra- and inter-assay coefficients of variation were found to be less 9% and 10%, respectively.

Regarding the weight of testis, it was recorded at the end of the experiment, and the testis index was calculated according to the following formula:

$$\text{Testis index}=\left[\text{testis weight}\left(\text{g}\right)/\text{final body weight}\left(\text{g}\right)\right]\times 100$$

### Estimation of testicular total protein

The testes'protein content was measured according to Lowry, et al. [30] procedure with bovine serum albumin as the reference protein.

### Estimation of oxidative stress parameters

Testicular content of MDA and activities of SOD and CAT were measured colormetrically, using commercially available kits (Bio-diagnostics, Dokki, Giza, Egypt) and the procedure was carried out according to the manufacturer’s instructions. The measuring of the MDA concentration depends on a reaction done in acidic medium for 30 min between thiobarbituric acid (TBA) and MDA at 95 ◦C. This reaction forms a thiobarbituric acid reactive product. Concerning CAT, it firstly reacts with H_2_O_2_ (known amount), then after one minute, the reaction is stopped using the catalase inhibitor. The residual H_2_O_2_ reacts with 3,5-dichloro-2-hydroxybenzene sulfonic acid (DHBS) and 4-aminophenazone (AAP) in the presence of peroxidase (HRP), to form a chromophore with color intensity that is inversely proportional to the amount of catalase in the original sample. The principle of SOD assay mainly relies on an inhibitory reaction catalyzed by SOD for the phenazine methosulphate (PMS)-mediated reduction of nitroblue tetrazolium dye (NBT). The intensity of the resultant color was measured using Unicam 8625 UV/V spectrophotometer (Cambridge, UK), at their respective wavelengths (MDA at 534 nm, CAT at 510 nm, and SOD at 560 nm).

### Estimation of inflammatory markers

Testicular contents of IL-1β and PPAR-γ were assessed by ELISA technique via rat-specific IL-1β kit (Cat# ab100768, Abcam, Cambridge, MA, USA) and PPAR-γ kit (Cat# E-EL-R01056, Elabscience®, Texas, USA) according to the manufacturer’s instructions. The assay method was briefly clarified in a previous section (estimation of serum testosterone). For IL-1β, results are expressed as pg/mg; the intra- and inter-assay coefficients of variation were found to be less than 10% and 12%, respectively. For PPAR-γ, results are expressed as ng/mg, the intra- and inter-assay coefficients of variation were found to be less than 6%.

### Histopathological examination

Testicular tissues were preserved in 10% formaldehyde for 72 h. The sections were dehydrated using a succession of ethanol treatments followed by xylene impregnation with molten paraffin for light microscopy. After that, paraffin wax was used to embed the samples.

To view the structure, tissues were cut into 5 μm sections and stained with hematoxylin and eosin (H&E). The slides were then inspected at magnifications of 100x, 200x, and 400x.

#### Evaluation of seminiferous tubules and Johnsen scoring quantitatively

The seminiferous tubules'characteristics were gauged using an ocular grid. Twenty profiles with tubular circular or nearly circular shapes were chosen at random from each species. The mean value was then computed at a 400 × magnification using the two perpendicular diameters of each cross-section of the seminiferous tubules. Using Image J software, the diameters of each seminiferous tubule lumen and the height of the epithelium were determined. Johnsen Score was used in this study to compare the spermatogenesis of the normal and irradiated groups [31].

### Immunohistochemical determination of NF-ĸB

Immunohistochemical (IHC) analysis was used for measuring NF-κB p65 (active form) expression in testis tissues. Paraffin-embedded testis tissue sections of 3 μm thick, were rehydrated first in xylene, followed by graded ethanol solutions. Afterwards, the slides were blocked for 2 h using 5% bovine serum albumin (BSA) in Tris buffered saline (TBS). Then, IHC analysis was performed using a standard streptavidin–biotin-peroxidase procedure. Testicular sections from all groups were incubated with rabbit anti-NF-κB p65 polyclonal antibody (Cat# RP-1638, Thermo Fisher Scientific, Waltham, MA, USA) (dilution 1:100). Next, rinsing was done thoroughly with TBS, and sections were incubated with a biotinylated goat anti-rabbit secondary antibody. This is followed by washing and then 30 min of incubation with horseradish-peroxidase-conjugated streptavidin solution. Finally, the sections were washed with TBS and visualized with 3,3’-diaminobenzidine containing 0.01% H_2_O_2_. The expression of NF-κB p65 was detected by measuring the intensity of the developed brown color using a Leica application for slide analysis (Leica Biosystems- by Germany).

### Statistical data analysis

Data were statistically analyzed using Graphpad® Prism, version 6. Continuous data is displayed as the mean ± SEM. Data normality was verified by the Kolmogorov–Smirnov (KS, p > 0.10) test, and normal distribution was found for all data. One-way analysis of variance (ANOVA) was used to compare multiple groups, followed by Tukey's post-hoc test. Probability values less than 0.05 were considered statistically significant. The semi-quantitative histological scoring of testis damage was statistically analyzed using the Kruskal–Wallis ANOVA with a Dunn's post hoc test for comparing the medians of different groups.

## Results

### Effect of α-tocopheryl succinate on serum testosterone and relative testis/body weight ratio

**Fig. 2:**
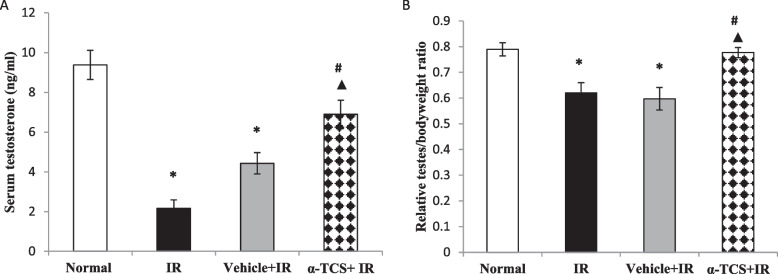
Effect of α-TCS on serum testosterone level (**A**) and relative testes body weight ratio (**B**) in radiation-induced testicular damage. Rats received α-TCS (200 mg/kg p.o.) for seven days and irradiation (6 Gy) was carried out on the fifth day. Results are expressed as the mean ± SEM (*n* = 6). ^*^: significantly different compared to normal group, ^**#**^: significantly different compared to irradiated (IR) group, ^▲^: significantly different compared to Vehicle + IR group

As shown in Fig. 2A, rats exposed to γ-radiation demonstrated a marked decrement in the serum testosterone level by 80% (*p* < 0.001) and displayed a significant decline in the relative testes/body weight ratio (Fig. 2B), reaching 21% (*p* < 0.05), as compared to normal group. The vehicle treated group did not exhibit any significant improvement in the serum testosterone level, as well as the relative testis/body weight ratio. On the other side, oral administration of α-TCS led to a nearly two-fold rise in the serum testosterone level, as compared to the IR group (*p* < 0.001) and returned the relative testes/body weight ratio to the normal level (*p* < 0.05). It was notable that α-TCS treated group showed a significant amendment in the serum testosterone level and relative testes/body weight ratio by 55% *(p* < 0.05*)* and 30% (*p* < 0.01), respectively, as compared to the group that received vehicle Fig. 2.

### Effect of α-tocopheryl succinate on the testicular oxidative balance

**Fig. 3:**
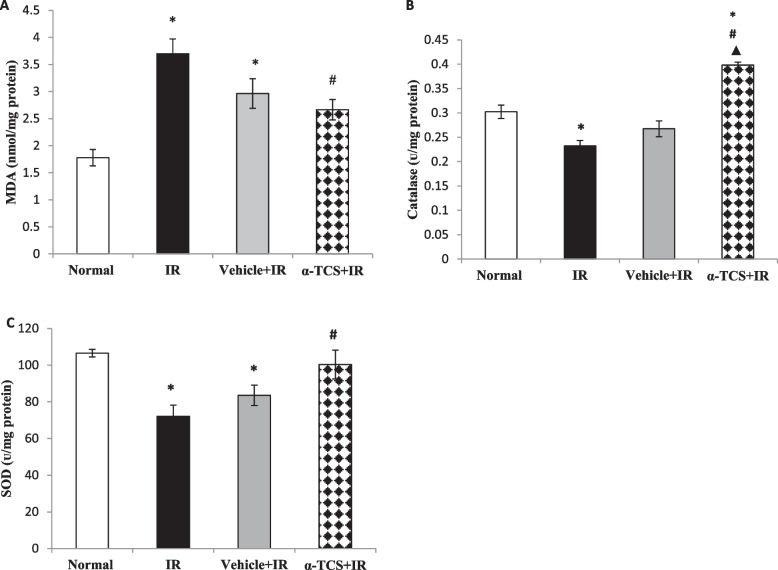
Effect of α-TCS on the testicular MDA content **(A)**, CAT activity (**B**), and SOD activity (**C**) in radiation-induced testicular damage. Rats received α-TCS (200 mg/kg p.o.) for seven days and irradiation (6 Gy) was carried out on the fifth day. Results are expressed as the mean ± SEM (*n* = 6). ^*^: significantly different compared to normal group, ^**#**^: significantly different compared to irradiated (IR) group, ^▲^: significantly different compared to Vehicle + IR group

Results of the current study revealed that exposure to γ-radiation induced a nearly one-fold increase in the MDA content (*p* < 0.001), which in turn suppressed the antioxidant enzymes CAT and SOD by 23% (*p* < 0.01) and 32% (*p* < 0.01), respectively, as compared to the normal group. Oral administration of α-TCS improved the testicular oxidative balance as it suppressed the MDA content by 30% (*p* < 0.05) and up-regulated the CAT and SOD activities by 71% (*p* < 0.001) and 39% (*p* < 0.05), respectively, as compared to the IR group. As anticipated, the IR group that received olive oil exhibited a minor alteration in oxidative stress markers, however this change was not statistically significant compared to the IR untreated group. Compared with the vehicle treated group, administration of α-TCS induced a notable change in the MDA and SOD contents, yet it showed a substantial rise in the CAT content by 71% (*p* = 0.001) (Fig. 3A, B, C).

### Effect of α-tocopheryl succinate on the testicular inflammatory response

**Fig. 4:**
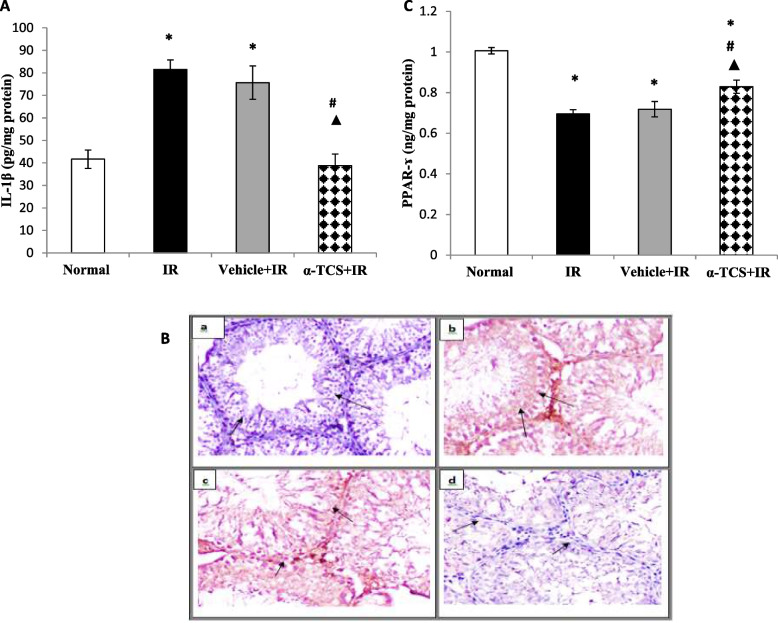
Effect of α-TCS on the testicular IL-1β content (**A**), NF-κB p65 expression (**B**) and PPAR-ɤ concentration (**C**) in radiation-induced testicular damage. The immunohistochemical examination of NF-κB p65 (× 400) in testicular tissue sections of normal group (**a**), irradiated (IR) group (**b**), vehicle + IR group (**c**) and α-TCS + IR group (**d**). Rats received α-TCS (200 mg/kg p.o.) for seven days and irradiation (6 Gy) was carried out on the fifth day. Results are expressed as the mean ± SEM (*n* = 6). ^*^: significantly different compared to normal group, ^**#**^: significantly different compared to irradiated (IR) group, ^▲^: significantly different compared to Vehicle + IR group

To explore the effect of α-TCS on the inflammatory response, testicular IL-1β content was estimated as a pro-inflammatory cytokine marker. Exposure to γ-radiation induced a nearly one-fold increase in the testicular IL-1β content, as compared to the normal group (*p* < 0.001). Treatment with α-TCS quenched the elevated IL-1β concentration by 52%, as compared to the IR group (*p* < 0.001), where it almost reached the normal level. In comparison with the rats that received vehicle, the α-TCS significantly suppressed the IL-1β content by 49% (*p* < 0.001) (Fig. 4A).

The expression of pro-inflammatory cytokines is highly relevant to the activation of intracellular signaling molecule NF-κB. Normally, NF-κB p65 subunit and the inhibitory protein IκB are complexed together in the cytosol. Upon activation, free p65 undergoes degradation and then translocated into the nucleus to stimulate the transcription of pro-inflammatory cytokines. Herein, the expression of p65 (active form) protein was estimated in testicular tissues by IHC. A negative expression was detected in the testicular tissues from the normal group, while higher immunostaining intensity was observed in testicular tissues of IR rats and those treated with vehicle. Oral administration of α-TCS markedly suppressed the p65 protein expression (Fig. 4B).

Among the pathways implicated in radiation-induced testicular dysfunction is the PPAR signaling pathway, which is considered to be an upstream regulator for NF-κB. To evaluate if the protective effect of α-TCS is associated with PPAR-dependent mechanism, the testicular concentration of PPAR-γ was estimated in the current study. Exposure to γ-radiation negatively altered the PPAR-γ protein expression by 31%, as compared to the normal group (*p* < 0.001). No discernible difference was found between the vehicle treated group and the IR non-treated group. On the contrary, treatment with α-TCS significantly enhanced the testicular PPAR-γ concentration by 20% (*p* < 0.05), as compared to the IR group and by 15% (*p* < 0.05), as compared to the IR group that received vehicle (Fig. 4C).

### Effect of α-tocopheryl succinate on the testicular histopathological changes

**Fig. 5:**
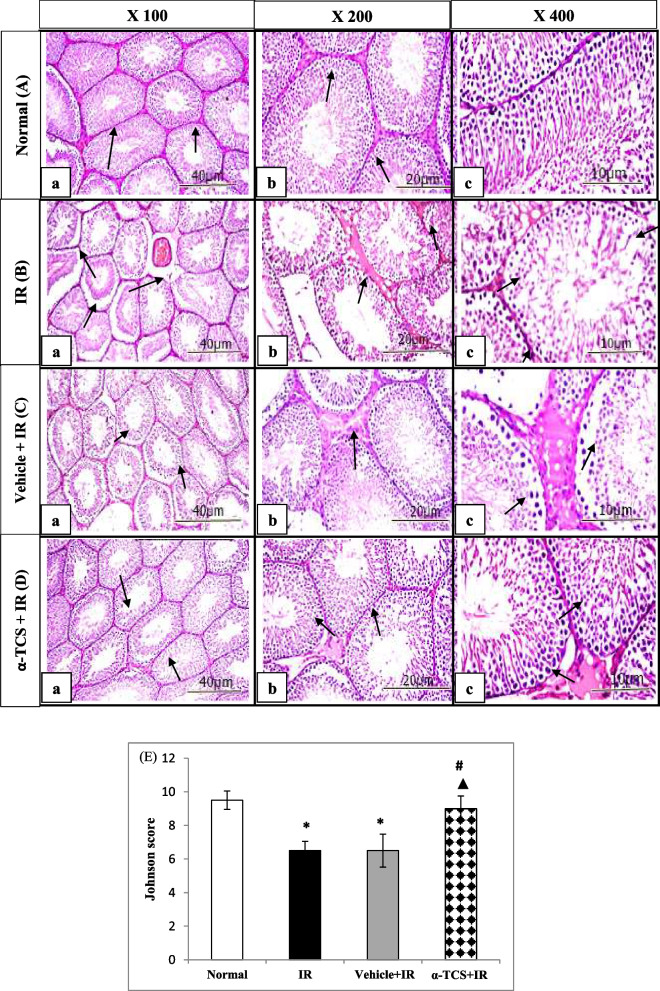
Photomicrographs of rat testicular tissue sections stained with H&E of normal (**A**), irradiated (IR) group (**B**), vehicle + IR group (**C**), and α-TCS + IR group (**D**) in radiation-induced testicular damage. Rats received α-TCS (200 mg/kg p.o.) for seven days and irradiation (6 Gy) was carried out on the fifth day. (**E**) Tissue sections were scored using Johnsen scoring system. Results are expressed as the median ± SEM (*n* = 6). *: significantly different compared to normal group, ^**#**^: significantly different compared to IR group, ^▲^: significantly different compared to Vehicle + IR group

The cross-sections for the normal control group showed that the interstitial tissue and tubules were intact. It also showed stratified germinal epithelium lining tightly packed seminiferous tubules, as well as Sertoli cells and spermatogonia resting on intact basement membranes. The spermatogenic cells were also shown to be made of the following layers: spermatogonia, spermatocytes, and spermatids. The narrow interstitium spaces between seminiferous tubules were made up of clusters of few Leydig cells. Moreover, agreeing with Johnsen scoring and histological outline of spermatogenesis was obvious (97.6%) Fig. 5A (a-b-c).

The seminiferous tubules of radiation-exposed group appeared atrophied, with widening of the interstitial spaces, eosinophilic edematous fluid, congestion of blood capillaries, and reduction of Leydig cells number with pyknotic nuclei. Disorganization of spermatogenic cells and folded basement membrane were found. Moreover, the spermatogenic cells showed vacuolations of their cytoplasm. Some spermatogenic cells appeared necrotic with pyknotic nuclei and hyperactivity of Sertoli cells were noticed. A significant decrement in the number of spermatogonia, primary and secondary spermatocytes, as well as, spermatids was shown in the seminiferous tubules. Seminiferous tubules had aberrant spermatids, a complete or partial reduction in spermatogenesis, loss of the germinal line, and decreased tubular diameter. Nearly a third of the spermatogenesis (33.8%) was seen Fig. 5B (a-b-c).

The testicular parenchyma of vehicle treated group showed that spermatogenic cells are disorganised within deformed, shrinking seminiferous tubules with vast interstitium gaps and eosinophilic edematous fluid. Also, the germinal epithelium thickness was reduced in the seminiferous tubules, and the lumina were large and empty. Moreover, different histological changes in spermatogenic cells were seen, including vacuolated cytoplasm, necrotic cells, and pyknotic nuclei. The number of spermatogonia was significantly reduced when compared with the normal control group. Vacuolation of Sertoli cells cytoplasm were also seen. Spermatogenesis was obvious according to Johnsen scoring and histological illustration (36.5%). These findings are similar to the IR group without any significant protection Fig. 5C (a-b-c).

On the contrary, testicular parenchyma of α-TCS treated group showed great protection of seminiferous tubules. Closely packed seminiferous tubules, approximately like those of the normal control group, were shown. The seminiferous tubules had regularly distributed spermatogenic cells, and spermatids were present in the typical tubular lumen. The spermatocytes, spermatids, and numerous layers of spermatogonia comprise the spermatogenic cells were also detected. Mild degrees of germinal cells swelling and interstitium edema were seen. Spermatogenesis was enhanced (79.7%), and the cells had a recognizable order. Some lesser effects of vacuolization were shown; however, edema was still detectable in the interstitial tissue Fig. 5D (a-b-c).

Semi-quantitative analysis revealed that exposure to γ-radiation significantly decreased the Johnsen scoring. Administration of olive oil to IR rats did not induce any significant improvement in Johnsen scoring. On the other side, administration of α-TCS before exposure to γ-radiation significantly ameliorated these histological perturbations, as compared to IR group, as well as the IR group received vehicle (Fig. 5E).

As shown in Table 1, quantitative analysis of the histomorphological parameters demonstrated that exposure to γ-radiation suppressed the number of spermatogenic cells (spermatogonia and spermatocytes) and significantly decreased the tube diameter, as compared to the normal group (*p* = 0.001). As compared to the IR group, no significant change was observed in any of these measurements in the vehicle treated group. Rats received α-TCS exhibited a significant elevation in the spermatogenic cells and the tube diameter, as compared to IR group and IR group received vehicle (*p* = 0.001), where a nearly normal histological appearance was observed. Notably, these outcomes suggest the advantageous impact of α-TCS in turning around the harmful impacts of radiation on the testicular function.

**Table 1:** Effect of α-tocopheryl succinate on different histological parameters in radiation-induced testicular damage

**Groups**	Normal	IR	Vehicle + IR	**α-TCS + IR**
Parameters				
Number of spermatogonia	73.33 ± 1.626	49.17^*^ ± 2.227	52.00^*^ ± 1.807	71.83 ^**#,▲**^ ± 1.302
Number of spermatids	117.5 ± 1.996	64.67^*^ ± 2.906	57.33^*^ ± 2.565	114.5 ^**#,▲**^ ± 2.930
Seminiferous tubules diameter (μm)	50.37 ± 1.032	33.60^*^ ± 0.608	31.81^*^ ± 0.930	47.72 ^**#,▲**^ ± 1.153
Lumen diameter (μm)	22.90 ± 0.748	17.23^*^ ± 0.583	17.83^*^ ± 0.591	21.72 ^**#,▲**^ ± 0.721

Rats received α-TCS (200 mg/kg p.o.) for seven days and irradiation (6 Gy) was carried out on the fifth day. Results are expressed as the mean ± SEM (*n*=6)

*significantly different compared to normal group

^#^significantly different compared to irradiated (IR) group

^▲^significantly different compared to Vehicle+IR group

## Discussion

As the advantageous use of ionizing radiation in diverse cancers escalates, so do the prospective health risks if not utilized appropriately. Whole body irradiation has a profound effect on reproductive function due to the radiosensitivity of the testes. Thus, male infertility and gonadal dysfunction could be serious long-term complications of radiotherapy [32]. Since the role of inflammatory response is closely implicated with oxidative stress in the pathogenesis of male infertility related to radiotherapy [33], there is a pressing need for the development of effective radioprotective compounds that possess both anti-inflammatory and antioxidant properties to safeguard male reproductive function. Hence, the possible protective effect of α-TCS, which has well-established antioxidant properties, was investigated focusing on the PPAR-γ/NF-κB/IL-1β pathway.

The testis is among the most radio-sensitive organs due to its significant proliferative ability [34], rendering it susceptible to free radical assault. Testicular spermatogenesis occurs in the germinal epithelium of seminiferous tubules, including successive mitosis and meiosis with very active proliferating activity [35]. Indeed, ionizing radiation could adversely alter the testis ultrastructure as well as spermatogenesis [33, 36]. Similarly, our work indicated that radiation exposure resulted in a considerable reduction in Johnsen's score, correlated with a drop in spermatogenic cells inside the germinal epithelium of atrophic seminiferous tubules, as seen by diminished tubule diameter. In males, testosterone is naturally produced from cholesterol via a variety of enzymatic mechanisms, primarily in the testicular Leydig cells and the adrenal glands [37]. Consequent to impaired spermatogenesis, testicular atrophy and lessened testosterone secretion were stated [7, 38]. This was further manifested in the current study as exposure to radiation instigated a significant decrease in serum testosterone level as well as the relative testis/body weight ratio. In addition to oxidative stress, studies have demonstrated that the toxicity of ionizing radiation is also attributed to the sustained inflammatory reactions followed by apoptosis of testicular cells, which led to a reduction in the total number of germ cells and the epithelial thickness of germinal cells [7, 36, 39].

Furthermore, our results demonstrated that α-TCS significantly improved the radiation-induced histological perturbations in which spermatogenesis was enhanced, and seminiferous tubules had regularly distributed spermatogenic cells together with an intact basement membrane. Moreover, α-TCS effectively returned the testosterone level and the relative testis/body weight ratio back to normal. Expectedly, the capability of α-TCS to maintain normal testicular structure extends beyond its antioxidant and anti-apoptotic effects [40, 41].

Particularly, as aforesaid, the radiation-mediated ROS overproduction is primarily responsible for the development of testicular harm and male infertility [1]. The formation of free radicals triggered a state of oxidative stress, confirmed by the end products of lipid, DNA, and protein peroxidation, which subsequently induced alterations in the redox system, enlightened by depletion of antioxidant enzymes [42, 43]. As observed in the current study, γ-irradiation induced an imbalance between pro-oxidant/antioxidant system, manifested by the remarkable rise in testicular content of MDA, the end product of lipid peroxidation, along with the significant decline in enzymatic activities of CAT and SOD. Such consequences could partially explain the observed deterioration in the testicular function of irradiated rats. Herein, the antioxidant potential of α-TCS was demonstrated through suppression of lipid peroxidation in parallel with restoring the antioxidant enzymatic activities of CAT and SOD. These findings align with prior researches that demonstrated the protective effects of Vitamin E against atrazine- or lead acetate-induced testicular damage, showing its capability in enhancing sperm quality and improving Leydig cells’ steroidogenic activity through the control of oxidative state [44, 45]. This could also be supported by a previous study that demonstrated the essential roles for vitamin E in testicular protection against cadmium toxicity, as it efficiently improved the disturbed antioxidant status, semen picture, and reproductive hormonal profile [40].

Besides oxidative stress, the inflammatory response was also regarded as one of the key elements contributing to testicular harm induced by radiation. The transcription factor NF-κB acts as a key modulator of inflammatory responses by inducing the expression of numerous pro-inflammatory genes, including those encoding cytokines and chemokines. In resting state, NF-κB p65 subunit is located in the cytosol complexed with the inhibitory protein IκB. Once activated, IκB undergoes phosphorylation by the IκB kinase (IKK) and subsequent degradation to produce free p65, which is then translocated into the nucleus and binds to its target genes, inducing transcription of pro-inflammatory cytokines [46]. Ionizing radiation has been shown to activate NF-κB either directly via breakage of the DNA double-strand [47] or indirectly through the generation of ROS that acts as a second messenger [48]. Likewise, our data indicated that NF-κB p65 expression levels were heightened post-irradiation exposure, showing that radiation caused ROS accumulation in the testis, leading to activation of NF-κB. This was followed by a significant rise in the inflammatory cytokine IL-1β content, which increased in accordance with the severity of inflammation.

Among the pathways linked to the regulation of NF-κB is the expression of PPARs that have been shown to play a significant role in the modulation of inflammatory responses by repressing the activity of NF-κB via agonist-dependent processes [49, 50]. Radiation exposure was reported to suppress the PPAR-γ expression, leading to an increase in oxidative imbalance and inflammatory response via activation of NF-κB [7, 51]. In agreement with the previous reports, our findings revealed that irradiation exposure suppressed PPAR-γ testicular content and exaggerated the expression of NF-κB p65 subunit, suggesting the implication of PPAR-γ/NF-κB cascade in radiation-induced testicular damage.

Although many attempts have been made to counteract the irradiation-induced testicular damage, there were no reports on the impact of α-TCS in such inflammatory cascade. In this regard, the current study investigates the effect of α-TCS on the PPAR-γ/NF-κB/IL-1β signaling cascade. With the aid of a specific agonist, PPAR-γ expression was reported to preserve testicular function through suppression of NF-κB expression [52]. Along this line, administration of α-TCS in the current study produced an anti-inflammatory effect, depicted by significant elevation in the PPAR-γ concentration along with the blockage of NF-ĸB activation, which subsequently led to downregulation in IL-1β level. The impact of α-TCS on the PPAR-γ/NF-κB cascade with the present pathological condition was not reported in the literature; yet, the study of Juretić, et al. [53] reported that dietary alpha- and gamma- tocopheryl supplementation in a high-fat-diet-fed murine model attenuated the expression of inflammatory markers in hepatic tissue through PPAR/NF-κB dependent mechanism. Moreover, several studies were carried out investigating the effect of α-TCS on cytokine release in different inflammatory models; the study of Gheita, et al. [54] postulated that α-TCS decreased the intestinal IL-1β content in radiation-induced gastrointestinal damage in mice. Additionally, in the study of Sarir, et al. [55], inflammatory cytokines were induced via high-intensity interval training and significantly diminished by administration of α-TCS in rats. Hence, our data indicate the crucial role of α-TCS as a PPAR-γ agonist in preserving testicular function partly through inhibiting NF-κB signaling and its downstream pro-inflammatory mediators expression.

## Conclusion

Collectively, radiation exposure negatively altered the testicular ultrastructure as verified by testicular atrophy, reduced spermatogenic cells, and diminished testosterone secretion. Moreover, radiation exposure disrupted the pro-oxidant-antioxidant equilibrium, resulting in oxidative stress, which is further sequenced with induced inflammatory cascade. Administration of α-TCS before and after γ-radiation exposure preserved the testicular architecture and ultrastructure, while restoring the testosterone hormonal level. As anticipated, α-TCS hampered the oxidative stress status and up-regulated the antioxidant enzymes SOD and CAT. Besides being a well-known antioxidant substrate, α-TCS effectively suppressed the inflammatory cytokine IL-1β, which prompted the current research to consider its anti-inflammatory role with particular concern on the PPAR-γ/NF-κB dependent cascade. Successfully, treatment with α-TCS significantly elevated the PPAR-γ expression and suppressed the activation of NF-κB. To sum up, the current study reports the promising mitigating role of α-TCS against radiation-induced testicular damage in rats via PPAR-γ/NF-κB pathway.

## Limitations

In the meantime, the existing data indicate that α-TCS offers a protective effect against early radiation-induced damage; however, the late effects of radiation exposure and their pathophysiology may differ, which is considered a limitation of the study, along with its impact on sperm count, motility, and morphology. Therefore, additional long-term studies would be conducted to ascertain whether comparable enhancements might be observed in the delayed effects of radiation exposure.

## Data Availability

The data that support the findings of this study are available from the corresponding author upon request.

## Abbreviations

ANOVA: Analysis of variance
CAT: Catalase
ELISA: Enzyme linked immunosorbent assay
IL-1β: Interleukin-1β
IL-6: Interleukin-6
IR: Irradiated
MDA: Malondialdehyde
NCRRT: National Centre for Radiation Research and Technology
NF-κB: Nuclear factor-kappa B
PPAR-γ: Peroxisome proliferator-activated receptor-γ
ROS: Reactive oxygen species
SOD: Superoxide dismutase
TNF-α: Tumor necrosis factor-alpha
WBI: Whole body gamma-irradiation
α-TCS: Alpha-tocopheryl succinate
